# Hypercapnia Impairs ENaC Cell Surface Stability by Promoting Phosphorylation, Polyubiquitination and Endocytosis of β-ENaC in a Human Alveolar Epithelial Cell Line

**DOI:** 10.3389/fimmu.2017.00591

**Published:** 2017-05-23

**Authors:** Paulina Gwoździńska, Benno A. Buchbinder, Konstantin Mayer, Susanne Herold, Rory E. Morty, Werner Seeger, István Vadász

**Affiliations:** ^1^Department of Internal Medicine, Justus Liebig University, Universities of Giessen and Marburg Lung Center, German Center for Lung Research, Giessen, Germany; ^2^Max Planck Institute for Heart and Lung Research, Bad Nauheim, Germany

**Keywords:** carbon dioxide, epithelial sodium channel, sodium transport, ubiquitination, alveolar fluid clearance, alveolar epithelium, mitogen-activated protein kinase signaling

## Abstract

Acute lung injury is associated with formation of pulmonary edema leading to impaired gas exchange. Patients with acute respiratory distress syndrome (ARDS) require mechanical ventilation to improve oxygenation; however, the use of relatively low tidal volumes (to minimize further injury of the lung) often leads to further accumulation of carbon dioxide (hypercapnia). Hypercapnia has been shown to impair alveolar fluid clearance (AFC), thereby causing retention of pulmonary edema, and may lead to worse outcomes; however, the underlying molecular mechanisms remain incompletely understood. AFC is critically dependent on the epithelial sodium channel (ENaC), which drives the vectorial transport of Na^+^ across the alveolar epithelium. Thus, in the current study, we investigated the mechanisms by which hypercapnia effects ENaC cell surface stability in alveolar epithelial cells (AECs). Elevated CO_2_ levels led to polyubiquitination of β-ENaC and subsequent endocytosis of the α/β-ENaC complex in AECs, which were prevented by silencing the E3 ubiquitin ligase, Nedd4-2. Hypercapnia-induced ubiquitination and cell surface retrieval of ENaC were critically dependent on phosphorylation of the Thr615 residue of β-ENaC, which was mediated by the extracellular signal-regulated kinase (ERK)1/2. Furthermore, activation of ERK1/2 led to subsequent activation of AMP-activated protein kinase (AMPK) and c-Jun N-terminal kinase (JNK)1/2 that in turn phosphorylated Nedd4-2 at the Thr899 residue. Importantly, mutation of Thr899 to Ala markedly inhibited the CO_2_-induced polyubiquitination of β-ENaC and restored cell surface stability of the ENaC complex, highlighting the critical role of Nedd4-2 phosphorylation status in targeting ENaC. Collectively, our data suggest that elevated CO_2_ levels promote activation of the ERK/AMPK/JNK axis in a human AEC line, in which ERK1/2 phosphorylates β-ENaC whereas JNK mediates phosphorylation of Nedd4-2, thereby facilitating the channel–ligase interaction. The hypercapnia-induced ENaC dysfunction may contribute to impaired alveolar edema clearance and thus, interfering with these molecular mechanisms may improve alveolar fluid balance and lead to better outcomes in patients with ARDS.

## Introduction

Carbon dioxide (CO_2_) is formed as a by-product of cellular respiration and is eliminated from the body during breathing (1). In respiratory disorders that are associated with alveolar hypoventilation, retention of CO_2_ is often detected, which leads to elevated CO_2_ concentrations in the blood, also known as hypercapnia (2). For example, patients with severe acute respiratory distress syndrome (ARDS) frequently present with hypercapnia, which may be enhanced due to mechanical ventilation with low tidal volumes to minimize further ventilator-induced injuries to the lung (3). It is increasingly evident that the alveolar epithelium is capable of sensing of elevated CO_2_ levels, which initiate specific signaling signatures and alter the function of alveolar epithelial and other cells (2, 4). While some of these effects are anti-inflammatory, which may be beneficial in the context of excessive inflammation, others impair innate immunity, mitochondrial function, cellular repair, and alveolar epithelial barrier function, which are clearly detrimental in the setting of ARDS (5–10).

A fully functional alveolar epithelial barrier is crucial for maintaining optimal fluid balance and gas exchange in the lung (11, 12). In order to keep the alveolar space “dry,” excess alveolar liquid is reabsorbed from the air space into the interstitium by a well-characterized active sodium transport process in which Na^+^ enters the alveolar epithelial type I and type II cells through the apically located epithelial sodium channel (ENaC) and is subsequently pumped out basolaterally by the Na,K-ATPase. This creates a Na^+^ gradient, which drives paracellular movement of water leading to its clearance from the alveolar space (11, 12). Importantly, it has been clearly demonstrated that in most patients with ARDS alveolar fluid clearance (AFC) is impaired and that those patients with ARDS and impaired AFC the mortality is significantly higher than in ARDS patients with normal AFC (13).

Other than in the alveolar epithelium, ENaC molecules are expressed in the apical surface of various tight epithelia including kidney, colon, and respiratory airways where ENaC is located along the entire length of motile cilia and regulate osmolarity of the periciliary fluid (14). ENaC usually consists of three subunits (α [or δ, depending on the species, tissue, and cell type], β, and γ) (15–17). A functional ENaC complex requires at least one α- or δ-subunit, whereas the β- and γ-subunits are necessary for proper trafficking and activity of the channel (15, 16, 18). In line with this notion, mice lacking α-ENaC are unable to clear lung fluid from the alveoli and die nearly immediately after birth (19). The significance of β-ENaC in the regulation of channel activity has been highlighted in transgenic mice overexpressing this subunit in the lung (20). In these animals, an increase in alveolar epithelial Na^+^ uptake due to β-ENaC overexpression, probably by promoting trafficking of ENaC to the cell surface and enhancing channel activity, leads to lung dehydration and causes a CF-like phenotype. Cell surface abundance of ENaC is modified by the E3 ubiquitin ligase Nedd4-2, which by interaction with the PY motif, located at the C-termini of each subunit of the channel, promotes ubiquitination and subsequent clathrin*-*mediated endocytosis of the channel (21–23). Nedd4-2^−/−^ mice have enhanced ENaC expression and function; however, this genetic manipulation has lethal consequences (24). In contrast, overexpression of the ligase causes a decrease in ENaC density at the plasma membrane (PM) and reduces Na^+^ transport (25).

Previous studies have proposed the involvement of phosphorylation in the mechanisms regulating Nedd4-2 binding to ENaC (26, 27). For example, extracellular signal-regulated kinase (ERK), c*-*Jun N*-*terminal kinase (JNK), and recently also the cellular energy sensor, AMP-activated protein kinase (AMPK), have been described as potential modulators of the ENaC/Nedd4-2 interaction by phosphorylating the E3 ligase or the target (28–32). We have previously described that hypercapnia markedly impairs AFC and initiates a specific signaling pattern in the alveolar epithelium, including rapid activation of ERK, AMPK, and JNK and subsequent downregulation of the Na,K-ATPase (7, 8, 33). Considering the pivotal role of ENaC in AFR and that several kinases, which have previously been suggested to alter ENaC/Nedd4-2 interaction, are activated by elevated CO_2_ levels, in the current study we sought to determine whether ENaC is effected by hypercapnia and provide evidence that excess CO_2_ initiates ERK-mediated β-ENaC phosphorylation and AMPK/JNK-dependent activation of Nedd4-2 leading to an enhancement of β-ENaC polyubiquitination and, thus, to endocytosis of the ENaC complex form the cell surface. Since ENaC activity is essential for optimal lung fluid balance, the hypercapnia-induced alterations in ENaC cell surface stability may cause further aggravation of lung injury.

## Materials and Methods

### Cell Culture

Human epithelial A549 cells (ATCC, CCL 185) were grown in DMEM supplemented with 10% fetal bovine serum and 100 U/ml penicillin, 100 µg/ml streptomycin as previously described (8). Experiments were performed on subconfluent monolayers of cells. Cells were incubated in a humidified atmosphere of 5% CO_2_/95% air at 37°C.

### CO_2_ Exposure

A549 cells were treated with 40 or 120 mmHg CO_2_ (normocapnia and hypercapnia, respectively). Before each experiment, fresh solutions were prepared with DMEM-Ham’s F-12 medium and Tris base. The buffering capacity of the experimental media was modified by changing the initial pH using Tris base to obtain a pH of 7.4 at 40 and 120 mmHg CO_2_ (8). The desired CO_2_ concentrations and pH levels were obtained by equilibrating the experimental media overnight in a humidified chamber from BioSpherix Ltd. (NY, USA). The C-Chamber’s atmosphere was controlled with a PRO-CO_2_ Carbon Dioxide controller (Biospherix Ltd.). In the chamber, cells were treated with a pCO_2_ of 40 or 120 mmHg while keeping 21% O_2_ balanced with N_2_. Before and after CO_2_ exposure, pH, pCO_2_, and pO_2_ levels in the media were measured using a Rapidlab blood gas analyzer (Siemens, Erlangen, Germany).

### Plasmids, Constructs, Site-Directed Mutagenesis, Antibodies, and Inhibitors

pEYFP-C1-expressing α-ENaC was constructed by PCR amplifying α-ENaC gene using as a template pTNT-α-ENaC and oligonucleotide primers α-ENaC forward 5′-GAATTCAATGGAGGGGAACAAGCTGGAGG-3′ and α-ENaC reverse 5′-GGATCCCTTGTCATCGTCATCCTTGTAATCGGGCCCCCCCAGAGGAC-3′. The resulting amplicon was digested with *Eco*RI/BamH1 and ligated to the multiple cloning site (MCS) of pEYFP-C1 plasmid. The pEYFP-C1 vector contained the epitope-tag eYFP at the N-terminus and a FLAG-tag at the C-terminus. Thus, anti-GFP or anti-FLAG antibodies recognize the α-ENaC construct at a predicted size of 118 kDa, 1,073 amino acids [α-ENaC (90 kDa) plus YFP (27 kDa) and FLAG (1 kDa)]. pcDNA3.1V5/His expressing β-ENaC was constructed by PCR amplifying β-ENaC gene using as a template cDNA transcribed from total mRNA isolated from A549 cells and oligonucleotide primers β-ENaC forward 5′-CTCGGATCCACATGCACGTGAAGAAGTACCT-3′ and β-ENaC reverse 5′-GCACTCGAGGATGGCATCACCCTCACTGT-3′. The resulting amplicon was digested with Xho1/BamH1 and ligated to MCS of pcDNA3.1V5/His plasmid. Finally, *E. coli* DH5α were transformed using the constructed plasmid. Anti-V5 antibodies recognize the β-ENaC construct at a predicted size of 96 kDa, 872 amino acids [β-ENaC plus V5 at the C-terminus (1 kDa)]. pCMV-HA-C-expressing γ-ENaC was constructed by PCR amplifying γ-ENaC gene using as a template pTNT-γENaC and oligonucleotide primers γ-ENaC forward 5′-AGGCCCGAATTCATGGCACCCGGAGAGAAGAT-3′ and γ-ENaC reverse 5′-GTAGCCGGTACCGAGCTCATCCAGCATCTGGG-3′. The resulting amplicon was digested and ligated to MCS of pCMV-HA-C plasmid. The pCMV-HA-C vector contained the epitope-tag myc at the N-terminus and an HA-tag at the C-terminus. Anti-HA antibodies recognize γ-ENaC at a predicted size of 97 kDa, 881 amino acids [γ-ENaC plus myc (1 kDa) and HA (1 kDa)]. pRK5-HA-ubiquitin was a gift from Ted Dawson [Addgene 17608 (34)] and the pCI HA NEDD4L plasmid was a gift from Joan Massague [Addgene 27000 (35)]. Site-directed mutagenesis was used to perform point mutation of T899A in human Nedd4-2 using Quick Change Mutagenesis Kit from Stratagene (La Jolla, CA, USA) in accordance to the manufacturer’s instructions. The primer sequences were as follows: Nedd4-2 forward: 5′-ACTGCAGTTTGTCGCAGGGACATCGCGAG-3′, Nedd4-2 reverse: 5′ CTCGCGATGTCCCTGCGACAAACTGCAGT-3′. Immunoblot analysis of epitope-tagged ENaC expressed in A549 cells were performed with a mouse anti-GFP antibody from Roche (Basel, Switzerland), a mouse anti-HA antibody (Covance, Princeton, NJ, USA), a mouse anti-V5 antibody and a mouse antibody against transferrin receptor used as loading control of biotinylated proteins from Invitrogen (Waltham, MA, USA; Figure S1 in Supplementary Material). A rabbit antibody directed against β-actin was used as loading control of cytoplasmic ENaC and was purchased from Sigma Aldrich (Saint Louis, MO, USA). The inhibitor of AMPK, Compound C, was from Merck Millipore (Darmstadt, Germany). The inhibitor of MEK, U0126 was from Promega (Fitchburg, WI, USA). siRNA against AMPK-α1 and Nedd4-2 and scrambled siRNA control were purchased from Santa Cruz Biotechnology (Dallas, TX, USA).

### Transient Transfection

A549 cells were transiently transfected with eYFP-α-ENaC, β-ENaC-V5, HA-ubiquitin, HA-Nedd4-2 wild type, or mutant by using nucleofection, as previously described (36). Briefly, cells were resuspended in 100 µl of the nucleofection solution SF (Lonza, Cologne, Germany), and 4–6 µg of DNA was added. Cells were placed in a cuvette and pulsed with the specified cell-type nucleofector program. After 10 min of incubation time, cells were cultured in DMEM supplemented with 10% FBS, 100 U/ml penicillin, and 100 µg/ml streptomycin. In some studies, 24 h before nucleofection with ENaC plasmids, cells were transfected with siRNA using Lipofectamine RNAiMAX (Invitrogen, Waltham, MA, USA) according to the instructions of the manufacturer. Experiments were performed 48 h later.

### Cell Surface Biotinylation

A549 cells were labeled for 20 min using 1 mg/ml EZ-Link *N*HS-SS-biotin (Pierce Biotechnology, Waltham, MA, USA) and lysed in lysis buffer (50 mM HEPES, 150 mM NaCl, 1 mM EGTA, 10% glycerol, 1% TritonX100). Surface proteins were pulled down with streptavidin-agarose beads from Pierce Biotechnology (Waltham, MA, USA) and analyzed by SDS-PAGE and immunoblot, as described previously (8).

### Ubiquitination Studies

A549 cells were transfected with ENaC plasmids (2 µg of each) and HA-ubiquitin (3 µg). In some studies, cells were co-transfected with a plasmid coding HA-Nedd4-2 (wild type or mutant T899A, 2 µg) or siRNAs (against AMPK-α1, Nedd4-2, or scrambled). Cells were exposed to 40 mmHg CO_2_ (Ctrl) or to 120 mmHg CO_2_ (CO_2_) for 15 or 30 min and lysed on ice in lysis buffer (50 mM HEPES, 150 mM NaCl, 1 mM EGTA, 10% glycerol, 1% TritonX100), containing a protease inhibitor cocktail from Roche. After lysing the samples, proteins were resolved in 8% polyacrylamide gel, transferred to nitrocellulose membrane (Optitran; Schleicher & Schuell, Dassel, Germany) using a semidry apparatus from Bio-Rad (Hercules, Berkeley, CA, USA). Membranes were blocked in 5% fat-free dried milk powder and immunoblotted with anti-GFP or anti-V5 to detect α- or β-ENaC, respectively. Films were overexposed to detect ENaC ubiquitin conjugates.

### Phosphorylation Experiments

Phosphorylation studies of ERK1/2, AMPK-α1, and c-Jun were performed using antibodies from Cell Signaling (Danvers, MA, USA). The anti-phospho-β-ENaC (T615) antibody was from Abcam (Cambridge, UK). A549 cells were treated with normal or elevated CO_2_ concentrations (40 or 120 mmHg, respectively) for the desired times, and then were washed with PBS twice and were lysed on ice in lysis buffer (50 mM HEPES, 150 mM NaCl, 1 mM EGTA, 10% glycerol, 1% TritonX100). Samples having the same amount of protein were resuspended in Laemmli sample buffer and boiled for 10 min at 98°C and immunoblotted with specific antibodies.

### Statistics

Data are presented as mean ± SEM and were analyzed using one-way analysis of variance (ANOVA) followed by a multiple comparison with the Dunnet test. *p* values of less than 0.05 were considered significant. GraphPad prism 6 (GraphPad software, San Diego, CA, USA) was used for the analysis and presentation of data.

## Results

### Acute Exposure to Elevated CO_2_ Levels Leads to ENaC Endocytosis by Promoting Polyubiquitination of β-ENaC

**Figure 1:**
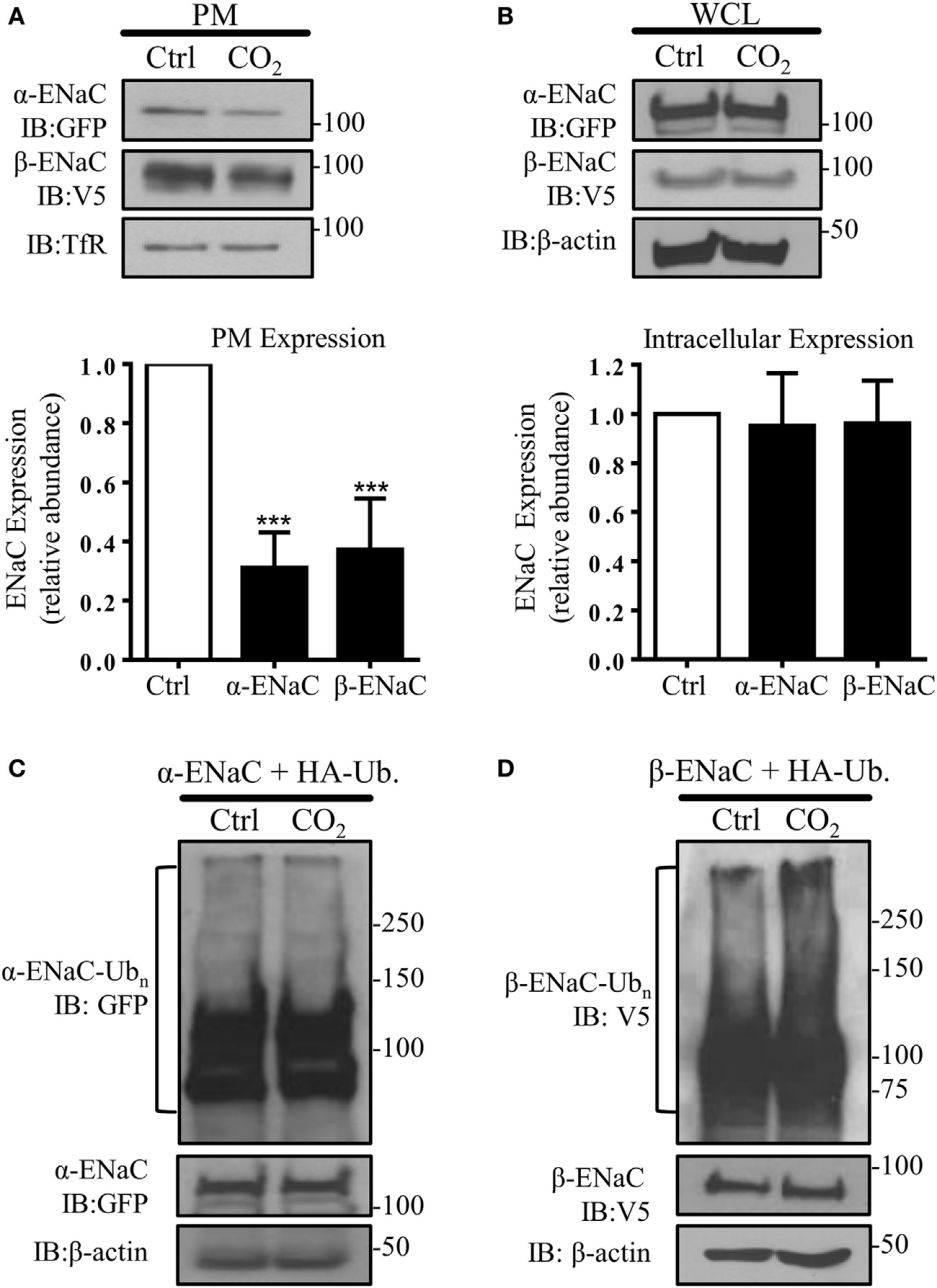
**Acute exposure to elevated CO_2_ levels leads to epithelial sodium channel (ENaC) endocytosis by promoting polyubiquitination of β*-*ENaC**. **(A)** A549 cells were co-transfected with α- and β-ENaC and were exposed to 40 mmHg CO_2_ (Ctrl) or 120 mmHg CO_2_ (CO_2_) for 30 min at a pH_e_ of 7.4. Plasma membrane (PM) proteins were determined by streptavidin pull-downs and immunoblotting with anti-GFP to detect α-ENaC and anti-V5 to detect β-ENaC. Representative immunoblots of α-, β-ENaC, and transferrin receptor (TfR) at the PM are shown. **(B)** A549 cells were co-transfected with α- and β-ENaC and were treated as described above. Protein abundance in whole cell lysate (WCL) was measured by immunoblotting. Representative immunoblots of α-, β-ENaC, and β-actin are shown. Bars represent mean ± SEM [*n* (number of independent experiments) = 3; ***p < 0.001]. **(C,D)**, A549 cells were co-transfected with ubiquitin containing HA-tag (HA-Ub) and α-ENaC **(C)** or β-ENaC **(D)** and were exposed to 40 or 120 mmHg CO_2_ for 15 min. Total ubiquitinated α-ENaC and β-ENaC was detected by immunoblots with anti-GFP or anti-V5 antibody.

To test whether high CO_2_ levels promote endocytosis of ENaC, A549 cells were co-transfected with plasmids encoding the human α- and β-subunit of ENaC and the PM abundance of these proteins was measured after exposure of cells to physiological (pCO_2_ 40 mmHg; normocapnia) or elevated (pCO_2_ 120 mmHg; hypercapnia) CO_2_ concentrations at a pH_e_ of 7.4 for 30 min. Exposure of cells to elevated CO_2_ levels decreased α- and β-ENaC cell surface abundance by approximately 60% (Figure 1A), whereas the total protein level remained unaffected (Figure 1B). To determine whether elevated CO_2_ levels lead to ubiquitination of either ENaC subunit, A549 cells were co-transfected with α- or β-ENaC and ubiquitin containing HA-tag (HA-Ub) and exposed the cells to 40 or 120 mmHg CO_2_ for 15 min. Whereas no ubiquitination of α-ENaC in response to hypercapnia was evident (Figure 1C), a marked increase in β-ENaC ubiquitination in total cell lysates was observed when cells were treated with elevated CO_2_ and immunoblotted with an antibody against V5 (Figure 1D). We detected a “polyubiquitin smear” above the molecular size of ENaC suggesting the presence of β-ENaC ubiquitin conjugates. This observation suggested that β-ENaC is a substrate of CO_2_-induced ubiquitination.

### Nedd4-2 Mediates the Hypercapnia-Induced ENaC Polyubiquitination and Endocytosis

**Figure 2:**
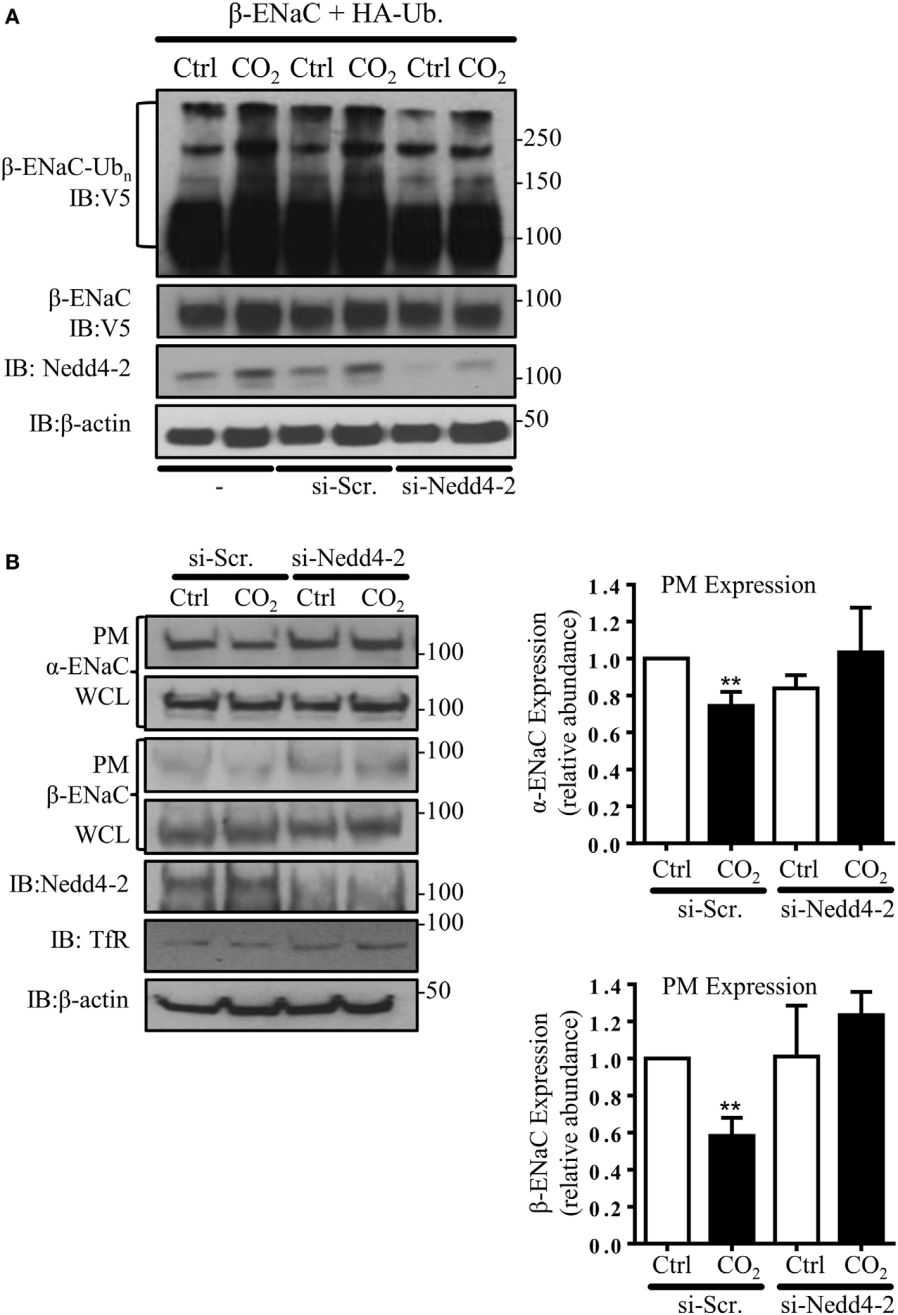
**Nedd4-2 mediates hypercapnia-induced epithelial sodium channel (ENaC) polyubiquitination and endocytosis**. **(A)** A549 cells were co-transfected with β-ENaC, HA-ubiquitin, and siRNA against Nedd4-2 or a scrambled siRNA (si-Scr.) and were treated with 40 or 120 mmHg CO_2_ for 30 min and β-ENaC polyubiquitinated isoforms were determined. **(B)** A549 cells were co-transfected with α-, β-ENaC, and siRNA targeting Nedd4-2 or scrambled siRNA. Biotinylated ENaC proteins were detected by immunoblotting. Representative immunoblots of α-, β-ENaC, and transferrin receptor (TfR) at the plasma membrane (PM) and total protein abundance [whole cell lysate (WCL)] of ENaC proteins, β-actin, and Nedd4-2 are shown. Bars represent mean ± SEM (*n* = 3; ** *p* < 0.01).

In subsequent studies, we silenced the endogenous Nedd4-2 with a specific siRNA to study whether Nedd4-2 mediates β-ENaC polyubiquitination during hypercapnia. Of note, the elevated CO_2_-induced ENaC ubiquitination was prevented by Nedd4-2 silencing (Figure 2A). To further test whether Nedd4-2 silencing altered ENaC PM stability, we measured cell surface ENaC abundance in A549 cells co-transfected with a scrambled siRNA (si-Scr.) or siRNA against Nedd4-2. Importantly, cells exposed to increased CO_2_ concentrations treated with siRNA targeting Nedd4-2 had increased ENaC α- and β-subunit density at the PM (Figure 2B). Thus, upon hypercapnia, Nedd4-2 targets β-ENaC, leading to polyubiquitination of the β-subunit of the channel, which results in decreased abundance of the α/β-ENaC complex at the cell surface.

### Hypercapnia Induces ERK1/2-Dependent Phosphorylation of β-ENaC at Thr615 and Downregulates Surface Abundance of the Channel by Facilitating β-ENaC Polyubiquitination and Endocytosis of the α/β-ENaC Complex

**Figure 3:**
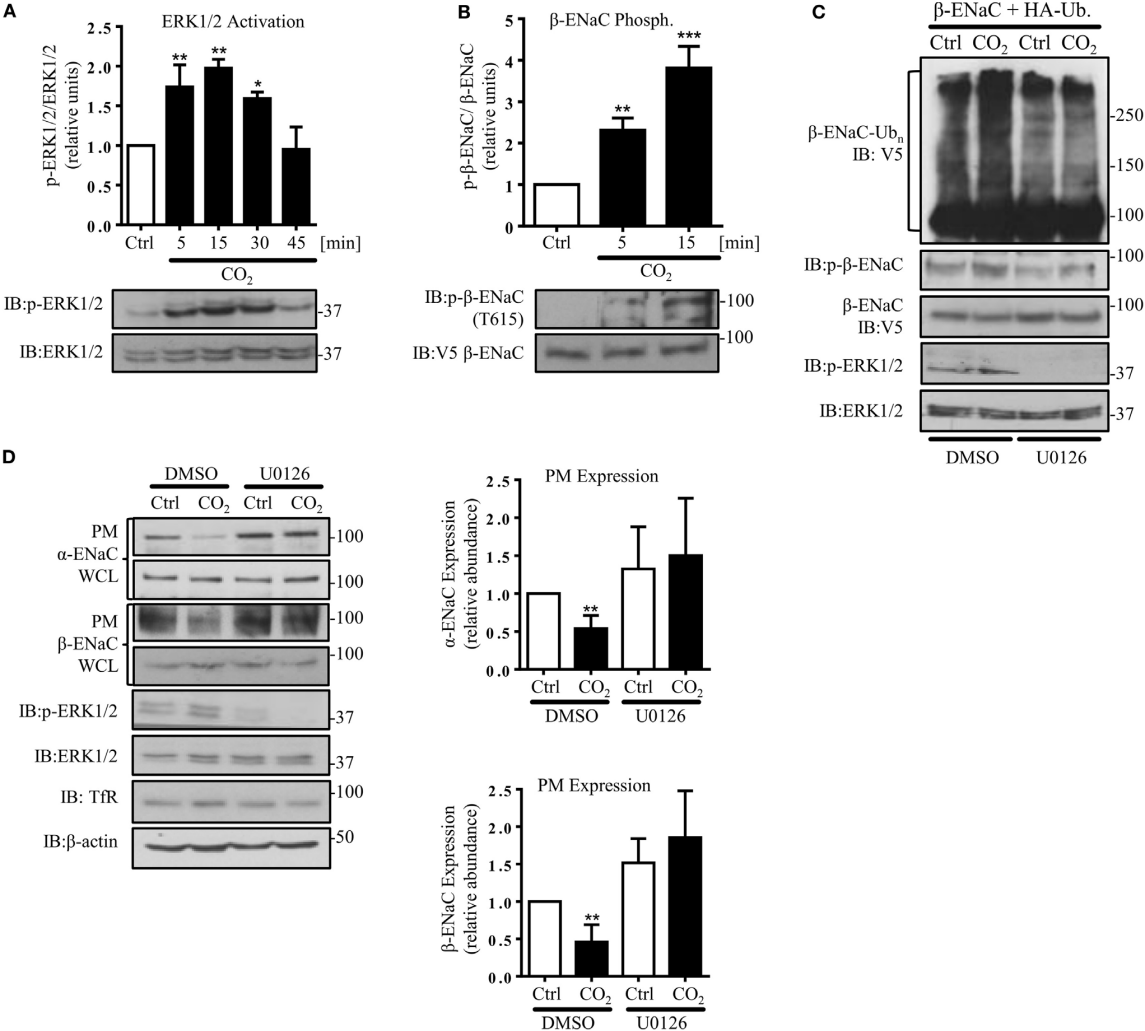
**Hypercapnia induces extracellular signal-regulated kinase (ERK)1/2-dependent phosphorylation of β-ENaC at T615 facilitating β-ENaC polyubiquitination and endocytosis of the α/β-ENaC complex**. **(A)** A549 cells were exposed to 40 mmHg CO_2_ (Ctrl) for 15 min or to 120 mmHg CO_2_ (CO_2_) for 5–45 min at a pH_e_ of 7.4. Phosphorylation of ERK1/2 and the total amount of ERK1/2 were measured. The graph represents the p-ERK1/2/ERK1/2 ratio. Representative immunoblots of p-ERK1/2 and total ERK1/2 are shown. **(B)** A549 cells were co-transfected with β-ENaC and were treated with 40 mmHg CO_2_ for 15 min or 120 mmHg CO_2_ for 5 and 15 min at a pH_e_ of 7.4. Phosphorylation of β-ENaC at T615 (p-β-ENaC) and total β-ENaC were determined by immunoblotting. Graphs represent p-β-ENaC/β-ENaC ratio. Representative immunoblots of p-β-ENaC and β-ENaC are shown. Values are expressed as mean ± SEM (*n* = 3; *p < 0.05; **p < 0.01). **(C)** A549 cells were transfected with β-ENaC, HA-ubiquitin, and were exposed to 40 mmHg CO_2_ or 120 mmHg CO_2_ for 30 min at a pH_e_ of 7.4 in the presence or absence of U0126 (10 µM, 30 min pretreatment). Total ubiquitinated β-ENaC was detected by immunoblotting with anti-V5 antibody. **(D)** A549 cells were co-transfected with α- and β-ENaC and were exposed to CO_2_ as described above. ENaC subunits at the plasma membrane (PM) were determined by biotin-streptavidin pull-downs and immunoblotting. Representative immunoblots of α- and β-ENaC at the PM, total protein abundance of epithelial sodium channel (ENaC) and p-ERK 1/2 are shown. Bars represent mean ± SEM (*n* = 3; *p < 0.05; ***p < 0.001).

In agreement with a previous report describing increased ERK1/2 activity in alveolar epithelial cells (AECs) exposed to hypercapnia (33), we found a rapid and transient phosphorylation of ERK1/2 in A549 cells exposed to elevated CO_2_ (Figure 3A). Moreover, ERK1/2 activation was paralleled by phosphorylation of β-ENaC at the Thr 615 residue (Figure 3B). To test whether ERK-dependent β-ENaC phosphorylation was sufficient to promote polyubiquitination of β-ENaC in response to high CO_2_, we co-transfected A549 cells with β-ENaC and HA-ubiquitin. Cells were pre-treated with the MEK (upstream of ERK) inhibitor U0126 and exposed to elevated CO_2_ concentrations for 30 min. Inhibition of ERK prevented phosphorylation and polyubiquitination of the ENaC β-subunit (Figure 3C). To further prove that the hypercapnia-induced ENaC cell surface retrieval is dependent on ERK1/2, A549 cells were co-transfected with ENaC plasmids, and exposed to normo- or hypercapnia as described above and observed that inhibition of ERK1/2 markedly increased the number of the ENaC molecules at the cell surface upon hypercapnia exposure (Figure 3D). Together, these data indicate that the hypercapnia-induced ERK1/2 activation promotes ENaC internalization by phosphorylation-dependent ubiquitination of the β-subunit of the channel.

### JNK1/2-Dependent Nedd4-2 Phosphorylation at Thr899 Facilitates β-ENaC Polyubiquitination and Endocytosis

**Figure 4:**
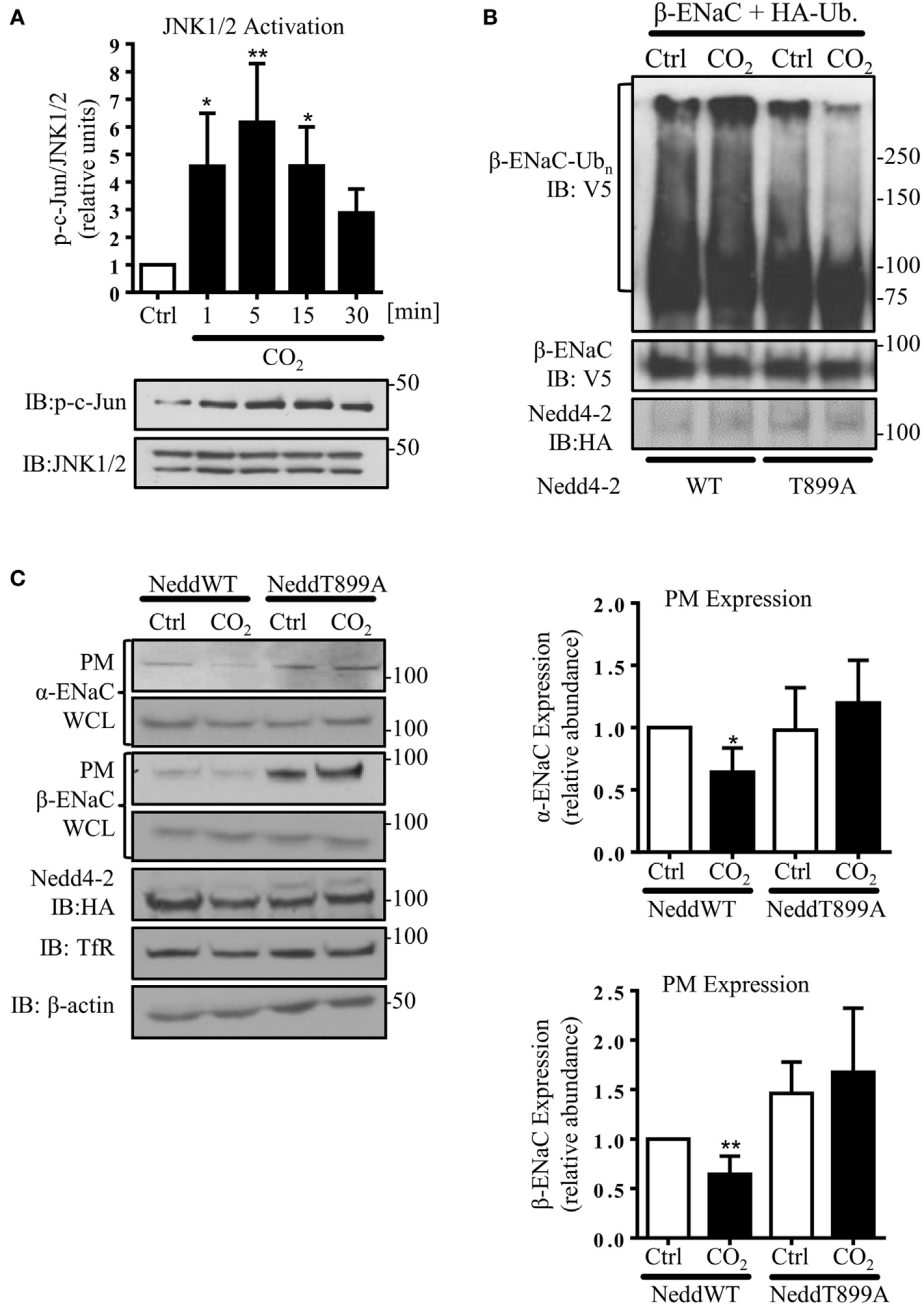

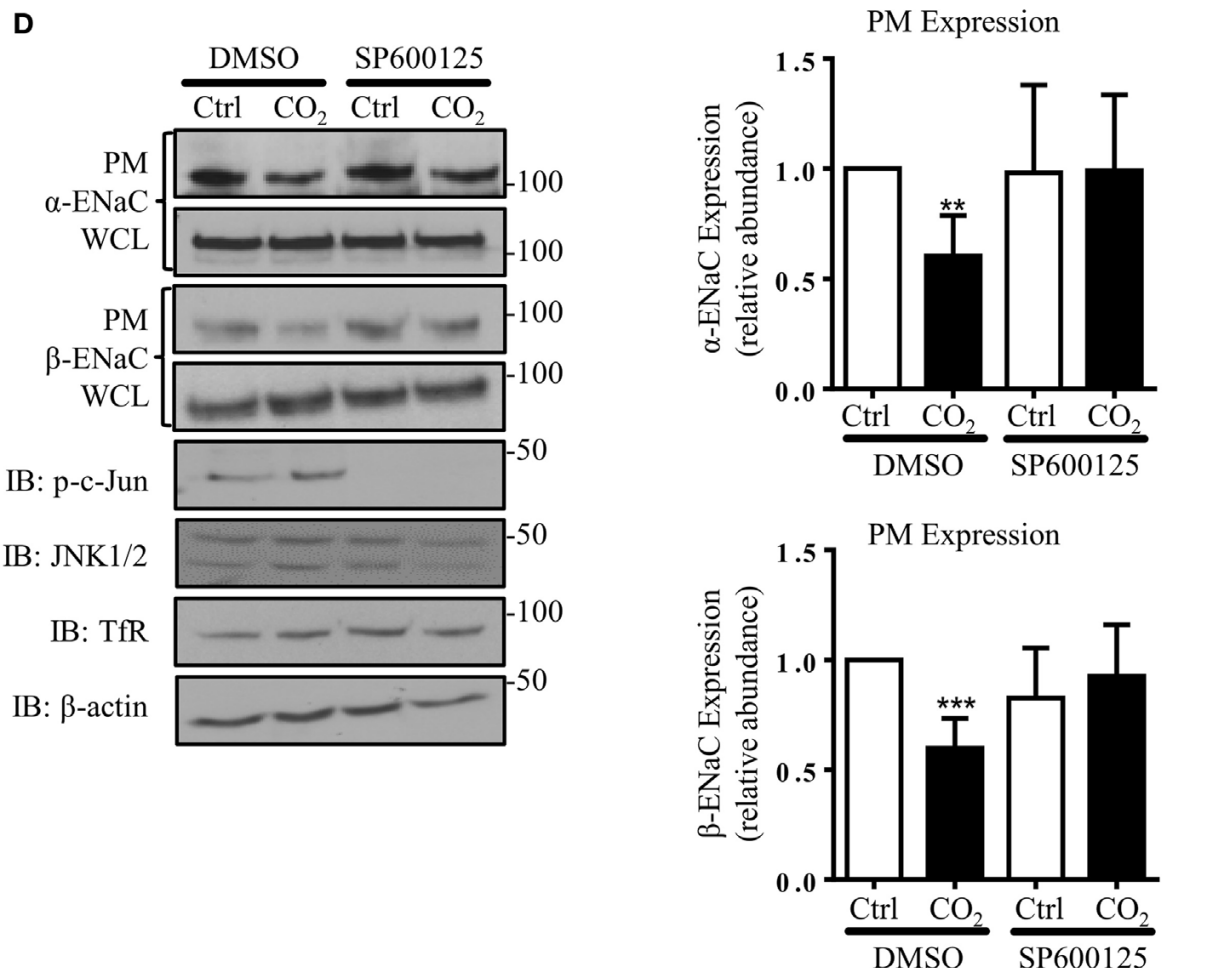
**c-Jun N-terminal kinase (JNK)1/2-dependent Nedd4-2 phosphorylation at Thr899 facilitates β-ENaC polyubiquitination and endocytosis**. **(A)** A549 cells were exposed to 40 mmHg CO_2_ (Ctrl) for 15 min or to 120 mmHg CO_2_ (CO_2_) for 1 to 30 min at a pH_e_ of 7.4 and the phosphorylation of c-Jun and the total amount of JNK1/2 were measured by immunoblotting. Graph represents the p-c-Jun/JNK1/2 ratio. Representative immunoblots of p-c-Jun and total JNK1/2 levels are shown. Values are expressed as mean ± SEM (*n* = 3; *p < 0.05; **p < 0.01). **(B)** A549 cells were co-transfected with β-ENaC, HA-ubiquitin, and HA-Nedd4-2 wild type (WT) or mutant (T899A). Cells were treated with 40 mmHg CO_2_ or 120 mmHg CO_2_ for 30 min at a pH_e_ of 7.4. Total ubiquitinated β-ENaC was detected by immunoblotting with anti-V5 antibody. **(C)** A549 cells were co-transfected with α- and β-ENaC and Nedd4-2 wild type or mutant and were exposed to CO_2_ as described above. Epithelial sodium channel (ENaC) at the plasma membrane (PM) was determined by biotin-streptavidin pull-downs and immunoblotting. Representative immunoblots of α- and β-ENaC at the PM, total protein abundance of ENaC, Nedd4-2, and β-actin are shown. Mean ± SEM (*n* = 3; *p < 0.05). **(D)** A549 cells were co-transfected with α- and β-ENaC exposed to 40 mmHg CO_2_ or 120 mmHg CO_2_ for 30 min at a pH_e_ of 7.4 in the presence or absence of SP600125 (25 µM, 30 min pretreatment). ENaC at the PM was determined by biotin-streptavidin pull-downs and immunoblotting. Representative immunoblots of α- and β-ENaC at the PM, total protein abundance of ENaC, p-c-Jun, JNK1/2, and β-actin are shown. Bars represent mean ± SEM (*n* = 5; **p < 0.01; ***p < 0.001).

Because JNK activation has been implicated in the CO_2_-induced signaling pattern in AEC, which led to inhibition of the Na,K-ATPase (7), we next investigated the effects of JNK phosphorylation in AEC exposed to hypercapnia on ENaC. Activity of JNK1/2 was assessed by phosphorylation of c-Jun, a downstream target of JNK1/2. In line with the previously published data, we observed a rapid and time-dependent JNK activation induced by hypercapnia, which returned to baseline within 30 min of exposure to elevated CO_2_ levels (Figure 4A). To further investigate whether increased activity of Nedd4-2 is crucial to decrease hypercapnia-induced ENaC cell surface abundance, we next mutated a single amino acid in the catalytic domain of Nedd4-2 (T899A). The Thr899 residue within the HECT domain of the E3 ligase has previously been reported to be involved in the Nedd4-2-mediated ubiquitination of α-ENaC (37). A549 cells were co-transfected with HA-Ub and HA-Nedd4-2 wild type (WT) or HA-Nedd4-2 mutant (T899) constructs and exposed to 40 or 120 mmHg CO_2_ for 30 min. Of note, we found that phosphorylation of Thr899 played a central role in the hypercapnia-induced ubiquitination of β-ENaC, as in A549 cells expressing the Nedd4-2 in which the Thr899 has been mutated to an alanine (T899A), which cannot be phosphorylated, the level of β-ENaC polyubiquitination significantly decreased (Figure 4B). To further investigate whether this decrease in the ubiquitination of β-ENaC due to the lack of phosphorylation at the Thr899 residue of Nedd4-2 correlated with an increase in ENaC cell surface stability, cell surface biotinylation studies were performed. Importantly, overexpression of the Nedd4-2 mutant (T899A) also prevented endocytosis of the α/β-ENaC complex during hypercapnia (Figure 4C). Moreover and further confirming the central role of Nedd4-2 phosphorylation at Thr899 in the ubiquitination and subsequent endocytosis of ENaC, we observed increased levels of ENaC proteins at the cell surface after overexpression of the Nedd4-2 T899A mutant. Finally and in line with the above described findings, pretreatment of A549 cells with the potent and specific JNK inhibitor, SP600125, also fully prevented the hypercapnia-induced endocytosis of ENaC (Figure 4D).

### Hypercapnia Induces ENaC Endocytosis by ERK1/2-Dependent AMPK-α1 Activation

**Figure 5:**
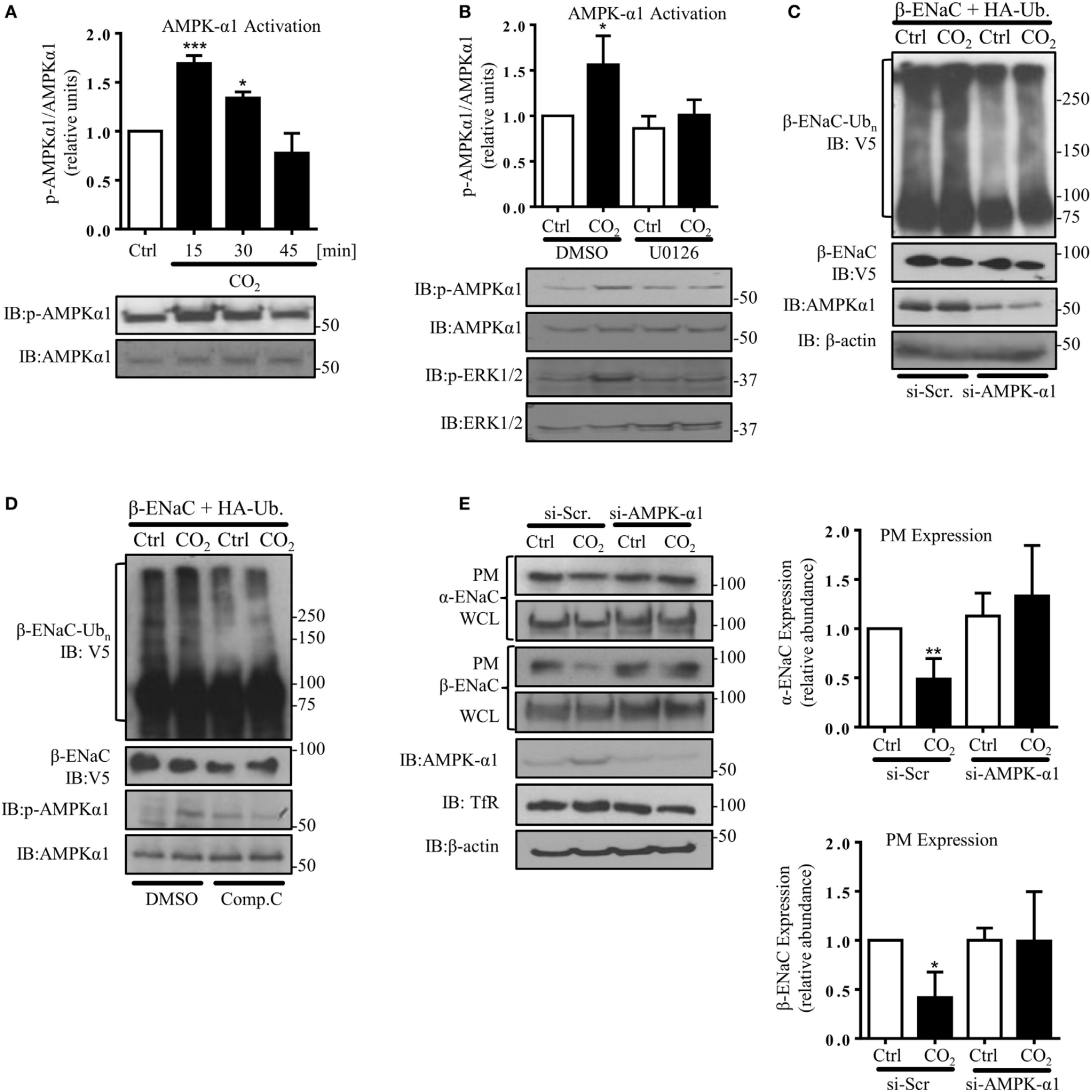
**Hypercapnia induces epithelial sodium channel (ENaC) endocytosis *via* extracellular signal-regulated kinase (ERK)1/2-dependent AMPK-α1 activation**. **(A)** A549 cells were exposed to 40 mmHg CO_2_ (Ctrl) for 15 min or to 120 mmHg CO_2_ (CO_2_) for 15–45 min at a pH_e_ of 7.4. The phosphorylation of AMPK-α1 at Thr172 and the total amount of AMPK-α1 were measured by immunoblotting. Graph represents the p-AMPK-α1/AMPK-α1 ratio. Representative immunoblots of p-AMPK-α1 and total AMPK-α1 are shown. **(B)** A549 cells were treated with 40 mmHg CO_2_ (Ctrl) or with 120 mmHg CO_2_ (CO_2_) for 15 min at a pH_e_ of 7.4 in the presence or absence of 10 µM U0126 (30 min pretreatment). Phosphorylation of AMPK-α1 at Thr172, p-ERK1/2, and the total amount of both proteins were determined by immunoblotting. Graph represents the p-AMPK-α1/AMPK-α1 ratio. Representative immunoblots of p-AMPK-α1, p-ERK1/2 and total level of AMPK-α1 and ERK1/2 are shown. Values are expressed as mean ± SEM (*n* = 3; *p < 0.05; ***p < 0.001). **(C)** A549 cells were co-transfected with β-ENaC, HA-ubiquitin, and siRNA targeting AMPK-α1 or a scrambled siRNA and were treated with 40 or 120 mmHg CO_2_ for 30 min. β-ENaC polyubiquitinated isoforms were determined with anti-V5 antibody. Representative immunoblots of β-ENaC, AMPK-α1, and β-actin are shown. **(D)** A549 cells were co-transfected with β-ENaC and HA-ubiquitin and were treated with 40 mmHg CO_2_ or 120 mmHg CO_2_ for 30 min at a pH_e_ of 7.4 in the presence or absence of compound C (20 µM, 30 min pretreatment). Total ubiquitinated β-ENaC was detected as described above. Representative immunoblots of β-ENaC, p-AMPK-α1, and total AMPK-α1 are shown. **(E)** Cells were co-transfected with α- and β-ENaC and siRNA targeting AMPK-α1 or a scrambled siRNA and exposed to 40 mmHg CO_2_ or 120 mmHg CO_2_ for 30 min at a pH_e_ of 7.4. Biotinylated ENaC proteins were detected by immunoblotting. Representative western blots of α- and β-ENaC at the plasma membrane (PM) and total protein abundance of ENaC, p-AMPK-α1, and AMPK-α1 are shown. Bars present mean ± SEM (*n* = 3; *p < 0.05; **p < 0.01).

AMP-activated protein kinase, which has been shown to activate Nedd4-2 and inhibit ENaC (29), has also been described as one of the central mediators of the hypercapnia-induced alveolar epithelial dysfunction and a downstream target of ERK upon CO_2_ exposure (8, 33). Furthermore, we have previously observed that AMPK activates JNK1/2 in AEC when exposed to elevated CO_2_ levels (7). In line with these previously published observations, we measured a rapid and transient phosphorylation of AMPK-α1 in A549 cells exposed to hypercapnia (Figure 5A), which was dependent on activation of ERK (Figure 5B) and upstream of JNK (Figure S2 in Supplementary Material). To determine whether activation of AMPK was necessary for the hypercapnia-induced polyubiquitination of β-ENaC, A549 cells were co-transfected with β-ENaC, HA-ubiquitin, and a specific siRNA against AMPK-α1 (or a scrambled siRNA) and were exposed to normal or elevated CO_2_ levels and observed a significant decrease in polyubiquitination of β-ENaC after CO_2_ exposure (Figure 5C). As a second approach, endogenous AMPK was inhibited by compound C after co-transfection of A549 cells with β-ENaC and HA-ubiquitin. Similar to our data that we obtained with AMPK silencing, exposure of the cells to hypercapnia in the presence of the inhibitor markedly decreased the hypercapnia-induced β-ENaC polyubiquitination (Figure 5D). To further confirm the role of AMPK-α1 in the CO_2_-induced downregulation of ENaC, we transfected A549 cells with α- and β-ENaC and exposed to elevated CO_2_ levels for 30 min in the presence or absence of the above mentioned siRNA against AMPK-α1 (Figure 5E) or compound C (Figure S3 in Supplementary Material) and observed that silencing or inhibition of AMPK stabilized ENaC proteins at the PM upon hypercapnia. Taken together, these latter studies suggest that AMPK by activation of JNK and subsequent phosphorylation of Nedd4-2 plays a central role in the hypercapnia-induced ubiquitination and endocytosis of ENaC.

## Discussion

In the present study, we show that elevated CO_2_ levels initiate a specific signaling pattern leading to ubiquitination-mediated retrieval of ENaC from the PM, thereby reducing cell surface abundance of the channel in a human AEC line. Hypercapnia is associated with a number of acute and chronic pulmonary diseases; however, it is not evident to what extent and by which mechanisms these elevated levels of CO_2_ may further impact on disease states. While hypercapnia and the associated acidosis have been shown to have anti-inflammatory effects, which might be advantageous at sites of excessive inflammation, recently, it has been clearly demonstrated that by impairing innate immunity, cellular repair, and alveolar epithelial function, elevated CO_2_ may play a role in the pathogenesis of ARDS and COPD (2, 5, 9, 10, 38). Furthermore, it is increasingly evident that patients with ARDS and COPD who present with hypercapnia have worse outcomes (3, 39, 40).

A major function of the alveolar epithelium is the clearance of excess alveolar fluid, thereby promoting effective gas exchange. This clearance is mediated by the concerted action of various sodium transporters, among which the apically located ENaC and the basolateral Na,K-ATPase have been identified as key players. Indeed, we have previously shown that the Na,K-ATPase is downregulated by hypercapnia; however, a potential regulation of ENaC by carbon dioxide has not been previously investigated. Various factors have been shown to affect ENaC cell surface abundance and function, including interleukin-1β, interleukin-4, transforming growth factor-β, LPS, or hypoxia (41–45), which similar to hypercapnia are often observed in patients with respiratory failure. As reducing hypercapnia without further damaging the lung is challenging, a better understanding of the molecular patterns initiated by elevated CO_2_ levels may help us to interfere with the deleterious signals, thereby rescuing or at least not further aggravating lung damage.

Ubiquitination is a posttranslational modification that regulates trafficking and stability of proteins (46). Numerous studies described that depending on the stimulus ENaC subunits may undergo multimono- or polyubiquitination leading to channel retrieval from the cell surface or degradation of ENaC (47–49). It is also well documented that the phosphorylation status of target molecules and the E3 ubiquitin ligase often play a pivotal role in the initiation of ubiquitination (50, 51). Previous findings established the significance of mitogen-activated protein kinase (MAPK) in the hypercapnia-induced impairment of AFC (7, 33). Moreover, it has also been described that ERK and JNK, two prominent members of the MAPK family, may alter the phosphorylation status of ENaC and the E3 ligase of the channel, Nedd4-2, respectively (31, 37).

Thus, we first investigated whether elevated CO_2_ concentrations affect ENaC cell surface stability by a mechanism involving ubiquitination of the channel and whether the MAPK pathway is involved in the hypercapnia-induced signaling events. Of note, a remarkable and rapid increase in polyubiquitination of β-ENaC and a significant reduction of the cell surface abundance of the α/β-ENAC complex were observed in AEC exposed to hypercapnia, as early as 30 min after CO_2_ exposure, suggesting that ENaC function is probably sensitive to changes in CO_2_ levels. In contrast, in the first half an hour after CO_2_ exposure, total intracellular levels of ENaC remained unchanged, suggesting that CO_2_ influenced the trafficking of the channel rather than protein degradation. Furthermore, no significant changes in the ubiquitination status of α-ENaC have been detected upon hypercapnic treatment, highlighting and further confirming the central regulatory role of β-ENaC in the trafficking of the channel (52).

Our data demonstrate that elevated CO_2_ levels cause a rapid and time-dependent ERK1/2 activation followed by phosphorylation of β-ENaC at the Thr615 residue. Moreover, genetic inhibition of Nedd4-2, the E3 ubiquitin ligase that drives ubiquitination of the various ENaC subunits (49), by a specific siRNA reduced β-ENaC polyubiquitination and prevented the hypercapnia-induced redistribution of α- and β-ENaC from the PM to the intracellular store, indicating a central role for Nedd4-2 in ENaC ubiquitination and endocytosis in AEC exposed to hypercapnia. Indeed, ERK1/2 has previously been described as a negative regulator of ENaC. For example, Eaton et al. showed that protein kinase C-δ drives ERK activation leading to ENaC internalization (30). Another study established that the ERK-mediated ENaC downregulation is promoted by phosphorylation β- and γ-ENaC, resulting in enhancement of Nedd4-2/ENaC interaction and thus, decreased Na^+^ transport (31). Therefore, hypercapnia by enhancing ERK activity promotes phosphorylation of the ENaC β-subunit, which may increase the affinity of the E3 ubiquitin ligase to ENaC.

We have previously shown that JNK is also implicated in CO_2_ responses and that phosphorylation of the kinase is required for the CO_2_-induced inhibition of the Na,K-ATPase in the alveolar epithelium (7). The significance of JNK in cellular adaptation to stress has been shown by several studies (53). Of note, the possible role of JNK in modulating Nedd4-2 activity and ENaC current has been reported in polarized kidney epithelial cells (28). Remarkably, this study also showed that the Thr899 residue in the HECT (homologous to the E6-AP carboxyl terminus) domain of Nedd4-2 may be phosphorylated by JNK1, which was required for ubiquitination of α-ENaC (28). To assess the potential involvement of JNK-mediated Nedd4-2 phosphorylation in the hypercapnia-induced downregulation of ENaC, we mutated Thr899 to Ala to prevent phosphorylation of the E3 ligase at this residue. Importantly, this point mutation largely prevented the CO_2_-induced polyubiquitination of β-ENaC although activation of JNK was evident and stabilized α- and β-ENaC at the cell surface. Thus, our data together with the previously published literature suggest that phosphorylation of Nedd4-2 by JNK at the Thr899 residue is critical for the hypercapnia-induced ubiquitination of β–ENaC, which drives endocytosis of the ENaC complex from the PM in AEC.

We have previously shown that AMPK, a cellular metabolic sensor that inhibits several ion transporters including the cystic fibrosis transmembrane conductance regulator, Na,K-ATPase, and ENaC, is rapidly activated by hypercapnia (8, 29, 54). Regarding the regulation of ENaC, it has been shown that chemical stimulation of AMPK by 5-aminoimidazole-4-carboxamide-1-beta-4-ribofuranoside inhibited ENaC activity in lung epithelial cells (55). Moreover, enhanced abundance of ENaC channels at the cell surface was reported in the distal airways in AMPK-α1^−/−^ mice (28). Interestingly, AMPK has also been reported to regulate Nedd4-2 activity (26, 29). In the current study, treatment of AEC with a specific siRNA against AMPK-α1 or an AMPK-α inhibitor, compound C markedly decreased CO_2_-induced β-ENaC polyubiquitination and endocytosis of α-, and β-ENaC, which is consistent with previous findings showing that in human embryonic kidney cells, AMPK activation promoted Nedd4-2/ENaC association (26). Furthermore, and in line with a previously published study (7), we also show that in the context of hypercapnia, AMPK is an upstream regulator of JNK. Thus, it is probable that the AMPK-regulated effects of CO_2_ on Nedd4-2 and ENaC are indirect and mediated by JNK. Moreover, although AMPK is an early element of the CO_2_-induced signaling pattern, its activation appears to be downstream of ERK upon hypercapnic exposure. This is of particular importance as ERK appears two have a dual role in the hypercapnia-induced downregulation of ENaC. On the one hand, it rapidly phosphorylates the β-subunit of the channel and by activating AMPK and JNK, it indirectly promotes phosphorylation of the E3 ligase Nedd4-2 as well. Of note, both of these phosphorylation events seem to be critically required for the CO_2_-induced ubiquitination and subsequent endocytosis of ENaC, probably by enhancing the association of the E3 ligase and the target molecule.

Our study has some clear limitations. Although we show a rapid activation of ERK and a subsequent phosphorylation of β-ENaC at the Thr615 residue, which is a known target of ERK, we have not investigated the potential rescue of β-ENaC ubiquitination or trafficking of the ENaC complex after preventing phosphorylation at this specific site. A mutation of this residue will be necessary to definitely prove that ERK-promoted phosphorylation of β-ENaC at this residue drives the downregulation of the channel upon hypercapnia. Moreover, the current study was performed exclusively in AECs and further *in vivo* investigations will be necessary to establish the role of the hypercapnia-induced signaling events identified in the current manuscript in ENaC-driven AFC and alveolar epithelial barrier dysfunction in an animal model of hypercapnic acute lung injury.

Taken together, our study shows for the first time that upon exposure to elevated CO_2_ levels, ENaC cell surface abundance is rapidly downregulated in a human AEC line by a specific, CO_2_-induced and ERK-, AMPK-, and JNK-mediated signaling pathway, which promotes phosphorylation of both β-ENaC and Nedd4-2, leading to ubiquitination of β-ENaC and subsequent internalization of the α/β-ENaC complex. This novel signaling pathway may contribute to the persistence of alveolar edema and thus, interfering with these molecular mechanisms may improve alveolar fluid balance and lead to better outcomes in patients with ARDS and hypercapnia.

## Author Contributions

Conception or design of the work: PG and IV; acquisition, analysis, or interpretation of data: PG, BB, KM, SH, RM, WS, and IV; drafting the work: PG and IV; revising it critically for important intellectual content: PG, BB, KM, SH, RM, WS, and IV. All the authors approved the final version of the manuscript and agreed to be accountable for all aspects of the work in ensuring that questions related to the accuracy or integrity of any part of the work are appropriately investigated and resolved.

## Conflict of Interest Statement

The authors declare that the research was conducted in the absence of any commercial or financial relationships that could be construed as a potential conflict of interest.
