# Supplementary material for: Hypercapnia Impairs ENaC Cell Surface Stability by Promoting Phosphorylation, Polyubiquitination and Endocytosis of β-ENaC in a Human Alveolar Epithelial Cell Line
Source: Front Immunol. 2017 May 23;8:591. doi: 10.3389/fimmu.2017.00591 (PMC5440515; doi:10.3389/fimmu.2017.00591)
Supplement: Supplementary file 1 [file Presentation_1.PDF]

Hypercapnia impairs ENaC cell surface stability by promoting phosphorylation, polyubiquitination and endocytosis of β-ENaC in a human alveolar epithelial cell line

Paulina Gwoździńska[1], Benno A. Buchbinder[1], Konstantin Mayer[1], Susanne Herold[1], Rory E. Morty[1,2], Werner Seeger[1,2], István Vadász[1]

[1]Department of Internal Medicine, Justus Liebig University, Universities of Giessen and Marburg Lung Center, Member of the German Center for Lung Research, Giessen, Germany
[2]Max Planck Institute for Heart and Lung Research, Bad Nauheim, Germany

Supplementary Figure Legends

Supplementary figure 1. *Plasma membrane protein abundance of ENaC subunits after co-transfection of A549 cells with α-, β- and γ-ENaC.* A549 cells were co-transfected with α-, β-, γ-ENaC and plasma membrane (PM) abundance of each subunit was determined as described in the Materials and Methods. Representative western blots are of duplicates are shown. Transferrin receptor (TfR) serves as loading control.

Supplementary figure 2. *AMPK is upstream of JNK in the hypercapnia-induced signaling pattern.* A549 cells were treated with 40 mmHg CO_2 (Ctrl) or with 120 mmHg CO_2 (CO_2) for 30 min at a pH_e of 7.4 in the presence or absence of the AMPK inhibitor, compound C (20 µM, 30 min pre-treatment). Phosphorylation of c-Jun (a downstream target of JNK1/2) and AMPK-α1 (at Thr172) and the total amounts of JNK1/2 and AMPK-α1 proteins were determined by immunoblotting. Graph represents the p-c-Jun/JNK1/2 ratio. Representative immunoblots of p-c-Jun, p-AMPK-α1, and total levels of JNK1/2 and AMPK-α1 are shown. Values are expressed as mean ± SEM. n = 3. *$p < 0.05$.

Supplementary figure 3. *Activation of AMPK is necessary for the hypercapnia-induced endocytosis of ENaC.* A549 cells were co-transfected with α- and β-ENaC and exposed to 40 mmHg CO_2 or 120 mmHg CO_2 for 30 min at a pH_e of 7.4 in the presence or absence of compound C (20 µM, 30 min pre-treatment). Biotinylated ENaC proteins were detected by immunoblotting. Representative western blots of α- and β-ENaC and transferrin receptor (TfR, loading control) at the plasma membrane (PM) and total protein abundance (whole cell lysate, WCL) of ENaC, p-AMPK-α1, AMPK-α1 and β-actin are shown. Bars present mean ± SEM. n = 3. *P < 0.05; **$p < 0.01$.

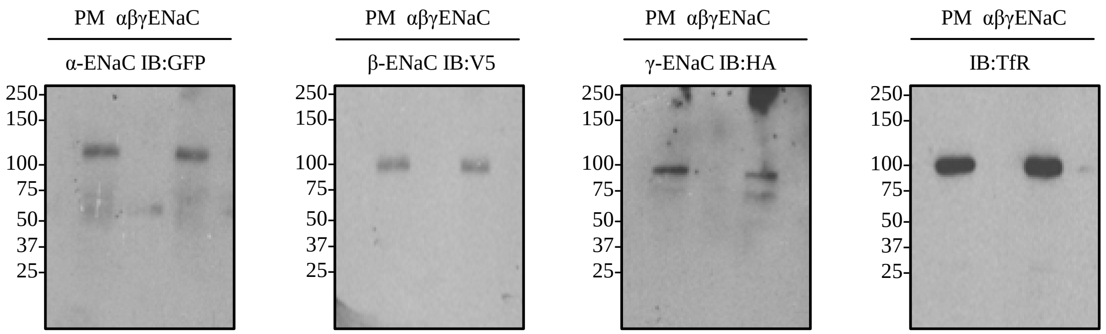

Supplementary figure 1

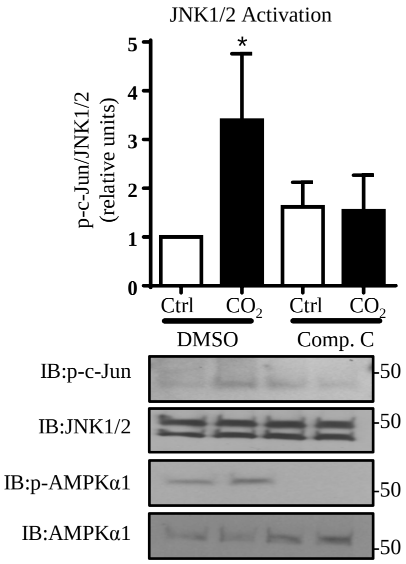

Supplementary figure 2

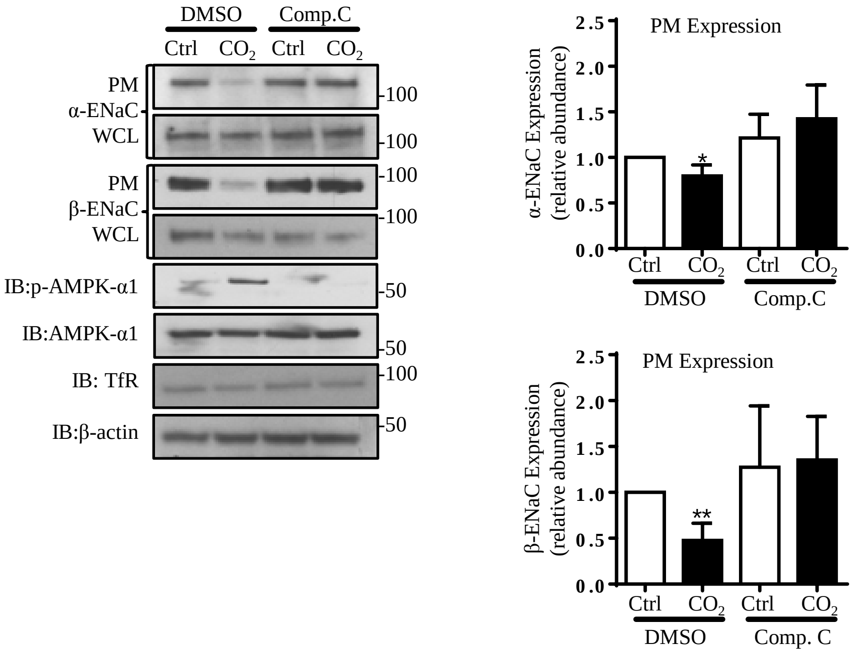

Supplementary figure 3
